# Addressing model discrepancy in a clinical model of the oxygen dissociation curve

**DOI:** 10.1098/rsta.2024.0213

**Published:** 2025-04-02

**Authors:** Gevik Grigorian, Victoria Volodina, Samiran Ray, Francisco Alejandro DiazDelao, Claire Black

**Affiliations:** ^1^ Department of Mechanical Engineering, University College London, London, UK; ^2^ Department of Mathematics and Statistics, University of Exeter, Exeter, UK; ^3^ Paediatric Intensive Care Unit, Great Ormond Street Hospital For Children NHS Trust, London, UK; ^4^ Clinical Operational Research Unit, University College London, London, UK; ^5^ University College London Hospitals, NHS Foundation Trust, London, UK

**Keywords:** model discrepancy, scientific machine learning, neural networks, symbolic regression, Gaussian processes

## Abstract

Many mathematical models suffer from model discrepancy, posing a significant challenge to their use in clinical decision-making. In this article, we consider methods for addressing this issue. In the first approach, a mathematical model is treated as a black box system, and model discrepancy is defined as an independent and additive term that accounts for the difference between the physical phenomena and the model representation. A Gaussian Process (GP) is commonly used to capture the model discrepancy. An alternative approach is to construct a hybrid grey box model by filling in the incomplete parts of the mathematical model with a neural network. The neural network is used to learn the missing processes by comparing the observations with the model output. To enhance interpretability, the outputs of this non-parametric model can then be regressed into a symbolic form to obtain the learned model. We compare and discuss the effectiveness of these approaches in handling model discrepancy using clinical data from the ICU and the Siggaard–Andersen oxygen status algorithm.

This article is part of the theme issue ‘Uncertainty quantification for healthcare and biological systems (Part 2)’.

## 1. Introduction

In recent years, there has been a growing effort to adopt mathematical models and simulations to support decisions across various fields [1]. Despite the advances in mathematical modelling and simulation, their limitations must be recognized and acknowledged. In this article, we discuss *model discrepancy*, also known as model error, model bias and structural uncertainty. This refers to the unavoidable difference between the real-world process of interest and the model representation used to study it. This discrepancy can arise due to various reasons. In particular, computational models are based on current scientific understanding, therefore, incomplete knowledge about the system being modelled can result in inaccuracies. In addition, simulation models require numerical approximation methods to produce their output, which will often lead to discrepancies between model output and observed values. Understanding and addressing model discrepancy is crucial in assessing the correctness, credibility and predictive power of the mathematical model when making high-stakes decisions.

Various approaches have been proposed to address model inadequacy of computational models. In weather forecasting and climate modelling, delta change and quantile mapping methods can be used to model the difference between the model predictions and the observations at the post-processing stage [2,3]. In uncertainty quantification (UQ), model discrepancy is also considered as part of inverse problems to obtain unbiased estimates of unknown model parameters [4], [5] proposed to define model discrepancy as an independent and additive term that represents the difference between the physical phenomena and the model representation. It is common to choose a stochastic process, namely a Gaussian Process (GP) to capture the model discrepancy since it is a flexible, non-parametric model that provides uncertainty estimates for the obtained predictions [5,6]. Most approaches propose to specify priors on model parameters, as well as model discrepancy, with the aim of performing a joint parameter and model discrepancy inference [4,5,7]. However, this type of inference is known to encounter non-identifiability issues, which can only be resolved by imposing stronger priors [4]. An alternative approach, which distinguishes between internal and external discrepancy, has been proposed by [8]. Internal model discrepancy can be quantified through experiments on the computational model, such as varying model parameters that are usually kept fixed. External model discrepancy directly relates to the limitations of the model and cannot be addressed as part of internal model discrepancy assessment. Interestingly, before this terminology was introduced by [8], [9] considered these types of discrepancies in their uncertainty analysis of a cosmological model, [10] proposed to use expert judgment and reified modelling to determine external model discrepancy [11]. In the majority of these approaches, the computational model is treated as a black box, where only the model’s inputs and outputs are observed. Consequently, we refer to these methods as black box approaches.

Occasionally, mathematical models can be partially known with some notable examples across various fields [12–14]. In the context of partially known models, recent developments in the field of scientific machine learning (SciML) have demonstrated the possibility of learning missing components of partially known systems from the observations. A popular SciML approach within the field of dynamical systems is universal differential equations (UDEs) [15], where the unknown components of a partially known model prescribed by a system of ordinary or partial differential equations (ODEs or PDEs) are set to be governed by a neural network. This hybrid structure is often called a grey box model [16] and is hereafter referred to as such. The available data are used to train the grey box model such that the embedded neural network captures the dynamics missing from the system. The trained network can subsequently be regressed down to mathematical expressions, providing insight into the missing physics, thereby transforming it into a learned model. This method has been used on simple dynamical systems [15], as well as more complex systems [17–20]. Although the mathematical model of interest in this paper is not a system of ODEs or PDEs, the general framework can still be applied in largely the same manner.

In this paper, we consider the mathematical model of the oxygen dissociation curve (ODC) of human blood, the Siggaard–Andersen (S.A.) algorithm [21], to illustrate the black box approach and methods from SciML, namely the grey box and learned models, in addressing model inadequacy. In clinical settings, clinicians can improve oxygen delivery to tissues using, for instance, supplemental oxygen or even mechanical ventilation. It is crucial to deliver the right amount of oxygen—low tissue oxygen levels may prevent energy production and lead to cell death, but high levels of tissue oxygen may also cause damage through reactive oxygen species [22]. In addition, high mechanical ventilation pressures can cause traumatic damage to lungs. Traditionally, intensive care unit (ICU) clinicians have used pO_2_ to guide treatment, although this is largely a guide to how much oxygen can cross the lung barrier into blood, rather than what can be delivered to tissues. More recently, pulse oximetry has gained common use to provide a continuous measure of haemoglobin oxygen saturations (SpO_2_) an estimate of the arterial haemoglobin oxygen saturations (SaO_2_). There is some uncertainty among clinicians about the optimum SpO_2_ value to target when making treatment decisions [23]. SpO_2_ (and SaO_2_) are markers of how much oxygen can be delivered to tissues, but how much is actually delivered depends on how strongly haemoglobin binds, and conversely, is willing to unload, oxygen. This property of haemoglobin, known as haemoglobin oxygen affinity, is defined by the ODC, which describes the relationship between haemoglobin oxygen saturations SO_2_ and the partial pressure of oxygen, pO_2_.

In practice, oxygen affinity is difficult to measure. Mathematical models such as the S.A. algorithm [21], have been adopted by the medical engineering industry to display affinity-based values in blood gas analyzers that are commonly used in ICU. Typically, these values have not found clinical use in decision making, due to clinician scepticism regarding model accuracy. A more accurate estimation of oxygen affinity would add information for more tailored clinical decisions regarding oxygen therapy.

The outline of this paper is as follows. In §2, we introduce a mathematical model of the ODC. §3 describes approaches to model discrepancy. Next, in §4, we illustrate our approaches with synthetic data study. In §5, we apply these methods to the real clinical data. §6 contains concluding remarks and discussion.

## 2. Oxygen-haemoglobin dissociation curve

The ODC describes the relationship between partial pressure of oxygen dissolved in blood (pO_2_) and the haemoglobin oxygen saturation (SO_2_), the proportion of haemoglobin that is saturated with oxygen relative to the total binding sites available. In short, the ODC expresses the affinity that haemoglobin, the major oxygen carrier in the blood, has for oxygen. In particular, the sigmoid shape of curve represents that oxygen binds to haemoglobin when the surrounding oxygen partial pressure is high, in the lungs, and dissociates from oxygen when partial pressure is low, in the tissue capillaries. In addition, the ODC can shift position due to changes in pH, temperature ( T ), 2,3-diphosphoglycerate (2,3-DPG) in red blood cells, partial pressure of carbon dioxide in the blood (PCO_2_) and haemoglobin variants (FCOHb, FMetHb, FHbF). In conditions of increased metabolic activity, where tissues require more oxygen, SO_2_ is relatively lower for a given pO_2_. This results in a rightward shift of the ODC, leading to greater oxygen dissociation from haemoglobin. The opposite effect occurs when the metabolic activity is low, with the haemoglobin affinity for oxygen increasing.

To construct the ODC and derive the affinity of haemoglobin for oxygen for an individual patient, we adopt the S.A. algorithm [21] together with patients’ recorded values. We note that 2,3-DPG, denoted as cDPG in the S.A. model, is difficult to measure clinically—indirect measurements of 2,3-DPG have been used in research laboratories and for quality control in blood banks [24]. There are various ways to estimate levels of cDPG. For instance, [25] proposed to use a nomogram to derive a cDPG level from observed pO_2_, PCO_2_, pH and p50, the value of partial pressure of oxygen when oxygen saturation of haemoglobin is 50%, [26] suggested to use p50_st_, the value of p50 under standard conditions for adult humans, to calculate cDPG value. However, it is unclear how this variable is derived in calculations performed in blood gas analyser machines.

A mathematical model representing the haemoglobin-dissociation curve can be written as


(2.1) $$\begin{array}{lcr} & y-y^{o}=(x-x^{o})+h\;\tanh(k^{o}(x-x^{o})), & \end{array}$$

where $k^{o}=0.5343$ , $y=\ln(\frac{s}{1-s})$ , $y^{o}=\ln(\frac{s^{o}}{1-s^{o}})$ , with $s^{o}=0.867$ , and $x=\ln(\frac{p}{p^{o}})$ with $p^{o}=7$ kPa and $x^{o}=a+b$ , $h=h^{o}+a$ , where $h^{o}=3.5$ and b=0.055×(T−37) . The actual position of the ODC in the coordinate system is represented by a Hill plot [27], which is given by y=(ln⁡(s/(1−s))) and x=ln⁡(p) that are used in the mathematical model, with s and p corresponding to the combined saturation of oxygen and carbon monoxide and the combined partial pressure of oxygen and carbon monoxide, respectively. To obtain s and p , we are required to perform the following transformations:


(2.2) $$\begin{array}{r} \begin{array}{r} \begin{array}{rl} p & =pO_{2}+\frac{pO_{2}}{SO_{2}}\times [\frac{\text{FCOHb}}{1-\text{FCOHb}-\text{FMetHb}}], \end{array} \end{array} \end{array}$$

(2.3) $$s=\frac{SO_{2}\times (1-\text{FCOHb}-\text{FMetHb})+\text{FCOHb}}{1-\text{FMetHb}}.$$

**Figure 1.**
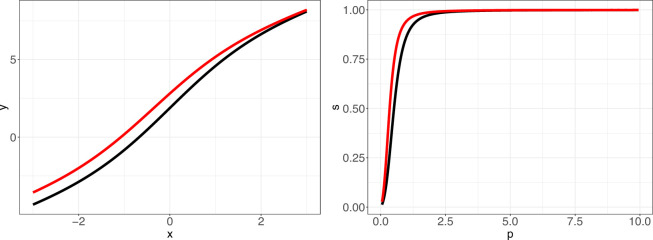
Oxygen dissociation curve (ODC): (*left panel*) logarithmic scale; (*right panel*) linear scale; black line corresponds to the reference ODC, red line represents the shift in the ODC due to alkalosis (blood pH increase).

The target used for training and inference in this work is s . The terms a and b reflect the ODC displacement from the reference position to its actual position. The term a describes the displacement at $37^{\circ }\text{C}$ , whereas b describes the additional displacement due to the patient temperature difference from $37^{\circ }\text{C}$ . The reference position of the ODC was chosen to be the one that corresponds to standard conditions for adult humans, namely: pH = 7.40, PCO_2_ = 5.33 kPa, FCOHb, FMetHb, FHbF = 0 and cDPG = 5 mmol/L. Figure 1 shows the ODCs on the logarithmic and linear scales. We can observe that a change in the a component of the model leads to the leftward shift of the ODC from its reference position. We chose to adopt the ODC on a linear scale to represent the results of our analysis since it is more interpretable in a clinical setting.

Typically, to derive the actual position of the ODC for a given patient, we must first calculate the shift of the reference curve at $37^{\circ }\text{C}$ due to changes in pH, partial pressure of carbon dioxide, variants of haemoglobin and cDPG represented by the term ac in equation (2.4):


(2.4) $$\begin{array}{lcr} & a=ac+a_{6}, & \end{array}$$

where the ac term is written as


$$\begin{array}{rl} ac & =a_{1}+a_{2}+a_{3}+a_{4}+a_{5},\\ a_{1} & =-0.88\times (\text{pH}-7.40),\\ a_{2} & =0.048\times \ln(\frac{{\text{PCO}}_{2}}{5.33}),\\ a_{3} & =-0.7\times \text{FMetHb},\\ a_{4} & =(0.3-0.1\text{FHbF})\times (\text{cDPG}/5-1),\\ a_{5} & =-0.25\times \text{FHbF}. \end{array}$$

In the second phase, we shift the curve further to pass through the known set of coordinates $\left(p_{0},s_{0}\right)$ obtained from performing the transformations in equations (2.2) and (2.3) from the patient’s measurements and adjust for the contribution of ‘unknown knowns’ ( $a_{6}$ ).

Based on the description above, we recognize that similar to other mathematical models, the ODC suffers from model inadequacy. Firstly, there is a lack of clarity among clinicians on how cDPG is estimated. In addition, it is known among the clinical community that the influence of 2,3-DPG changes with pH and temperature, which is not reflected in the model [28]. Secondly, a numerical method, such as the Newton–Raphson algorithm, can be used to calculate $a_{6}$ , which represents changes in ODC due to ‘unknown knowns’ and provides very limited interpretability for clinicians. In our analysis, instead of considering both terms $a_{4}$ and $a_{6}$ in equation (2.4), which can lead to serious non-identifiability issues, we assume that $a_{6}=0$ , and $a_{4}$ now represents the changes in ODC due to ‘unknown knowns’ that can include changes in 2,3-DPG.

## 3. Methods

Here, we present the black box model and approaches from SciML to address the model limitations discussed in §2. In particular, using a black box approach, we set $a_{4}=0$ within the S.A. model and aim to use a GP to account for the model’s limitations arising from its failure to account for changes in the ODC due to ‘unknown knowns’ when performing model-based inference regarding a patient’s oxygen affinity. On the contrary, the SciML approach aims to explicitly capture the behaviour of the missing component, $a_{4}$ , by constructing a grey box model wherein $a_{4}$ is defined as the output of a neural network and the remaining model structure is retained. The neural network is trained with observational data. To obtain the learned model, we regress the network down to mathematical expressions, which increases interpretability by providing insight into the dynamics of the system. The inputs to both the neural network and the GP are [pH, PCO_2_, FMetHb, T, p ].

### (a) Black box model and Gaussian process (GP)

We propose to treat a mathematical model as a function f that takes as input the parameter vector $x=(x_{1},x_{2},\ldots ,x_{p})\in {\mathbb{R}}^{p}$ and produces output f(x) . We define model discrepancy as the systemic difference between computational model predictions and the corresponding physical process of interest. Following [5], we choose to represent model discrepancy, denoted as δ(x) , as an additive term that depends on the input vector x . The relationship between the observation z and the model output f(x) is then given by


(3.1) $$\begin{array}{lcr} & z=f(x)+\delta (x)+e, & \end{array}$$

where e is the observation error term, modelled as Gaussian additive noise, i.e. $e∼N(0,\sigma_{e}^{2})$ . We further assume that all three terms in equation (3.1) are independent of each other. Suppose we have n observations of the physical system of interest, denoted by $z=\left(z_{1},z_{2},\ldots ,z_{n}\right)$ , associated with inputs $X=\left(x_{1},\ldots ,x_{n}\right)$ . The objective of model discrepancy inference is to estimate the discrepancy term δ(x) using observations of the physical system together with the computational model. We can then use the resulting updated model f(x)+δ(x) to perform inferences about the true physical process of interest. We note that contrary to [4,5,7], which considered calibration parameter estimation as part of the inference problem, we solely focus on model discrepancy inference.

We choose a stochastic process, namely the GP, to represent the model discrepancy. This is a class of flexible, nonparametric models that are capable of approximating an unknown function of interest [6]. GP also provides a measure of uncertainty about the obtained prediction, which is crucial for credibility assessment.

We specify a zero-mean GP prior for δ(⋅) with covariance function k(⋅,⋅) so that


(3.2) $$\begin{array}{lcr} & \delta (\cdot )∼GP(0,k(\cdot ,\cdot )), & \end{array}$$

with separable squared exponential covariance function:


(3.3) $$\begin{array}{lcr} & k(x,x^{\prime })=\sigma^{2}\exp\{-\sum_{i=1}^{p}(\frac{x_{i}-x_{i}^{\prime }}{\gamma_{i}}{)}^{2}\}, & \end{array}$$

where $\sigma^{2}$ and $\gamma =\left(\gamma_{1},\ldots ,\gamma_{p}\right)$ are a variance parameter and a vector of correlation length parameters, respectively. The variance parameter controls the scale of the model discrepancy, whereas the correlation length parameters determine how far apart x and $x^{\prime }$ need to be before δ(x) and $\delta (x^{\prime })$ become uncorrelated [4,29]. In particular, stronger correlation in model discrepancy for x and $x^{\prime }$ in the i th direction can be obtained with larger values of $\gamma_{i}$ , whereas the exact opposite holds for small values of $\gamma_{i}$ . We choose the squared exponential covariance function, a widely used kernel function for GPs [29]. We are interested in obtaining the posterior distribution of δ(x) . Similar to [4], we can integrate our prior knowledge about the model discrepancy by conditioning the process and its derivatives at pre-specified points. We demonstrate how this can be done in practice in §4 (see appendix (A.3) for computational details).

### (b) Grey box model and neural networks (NNs)

Artificial neural networks (NNs) are powerful nonparametric models, that are made up of neurons (a placeholder for a value) arranged in layers with connections between them, but can take a wide range of architectural forms depending on the specific task. The simplest version is known as a fully connected (or dense) network, where each neuron in a given layer is connected to each neuron in the next layer. The i th output of a fully connected network with one hidden layer, $\delta_{i}(x)$ , can be written as


(3.4) $$\begin{array}{lcr} & \delta_{i}(x)=b_{i}^{1}+\sum_{j=1}^{N_{1}}w_{ij}^{1}x_{j}^{1}(x),\;x_{j}^{1}(x)=\phi (b_{j}^{0}+\sum_{k=1}^{p}w_{jk}^{0}x_{k}), & \end{array}$$

where $w_{ij}^{l}$ and $b_{i}^{l}$ are components of weight and bias parameters for the l th layer, ϕ(⋅) is an activation function, and $N_{l}$ denotes the number of neurons in the l th layer (the width of the layer).

A grey box model can be constructed by defining the output of the NN as a component or multiple components within a mathematical model. To train the embedded NN, the grey box model is simulated to produce predictions, which are compared to ground truth values, or observations, in order to calculate a loss value. The objective is to update the weights and biases of the NN so as to minimize this loss value. The mean-squared error (MSE) [30] is a commonly used loss function for regression tasks and is used in this work.

Popular choices for the optimization are the Adam (adaptive moment estimation) [31] and BFGS (Broyden, Fletcher, Goldfarb and Shanno) [32–35] optimizers. Adam is efficient in moving the network parameters into a more favourable region, after which the BFGS optimizer (a quasi-Newton algorithm) is used, which utilizes second-order information about the loss function (the Hessian matrix) and is able to converge to a minimum efficiently. In [31] and [36], the details of the Adam and L-BFGS algorithms (respectively) are given.

### (c) Learned model with symbolic regression (SR)

Inferring mathematical expressions from the trained NN can provide insight into the underpinning mechanics of the system (as a nonparametric model is converted to an interpretable expression), and can also occasionally improve extrapolations, i.e. making predictions beyond the range of training data.

The sparse identification of nonlinear dynamics (SINDy) [37] and symbolic regression (SR) [38] are two popular choices for inferring mathematical expressions from measurement data. In this work, SR is used due to less prior knowledge requirements and its flexibility in learning more complex functions.

SR requires a set of unary operators (e.g. sin,cos,exp, etc.) and a set of binary operators (e.g. +,−,×,÷, etc.), specified by the user. Through a method known as genetic programming [38], the function space defined by the unary and binary operators is searched in order to find the expression that fits the data best. The fitness of each expression is determined by calculating an error measure (such as MSE) between the dynamics predicted by that expression and the target data. Through a series of processes known as **mutations**, **crossovers**, **tournaments** and **migrations**, new expressions are generated and those that fit the data best are the ones that survive.

The input and target data for SR are the inputs and outputs of the trained neural network, respectively. For a more detailed description of SR and its underpinning processes, see [39] and [40]. In this work, the Python package (with Julia back-end) PySR [41] is used to implement SR.

## 4. Synthetic data study

**Figure 2.**
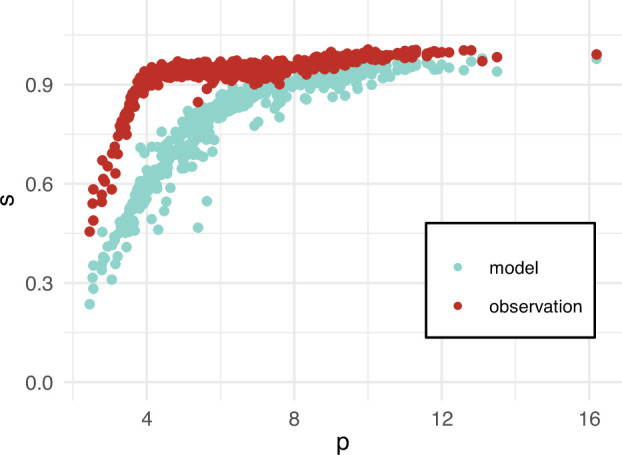
The model predictions with $a_{4}=0$ are represented by the blue points, while the observations obtained from a `ground truth' model with additive Gaussian noise are represented by the red points.

Initially, we conducted a synthetic data study to assess how our methods can address the inadequacies of a nested model like the S.A. model introduced in §2. We used this same model, referred to as the ‘ground truth’, with output s and inputs pH, PCO_2_, FMetHb, T and p , but specified $a_{4}=0.25\;\cos(2\pi \;\ln(p))-0.55$ in equation (2.4). The foetal haemoglobin (FHbF) is negligible and usually not recorded for adult patients, therefore, we set FHbF to zero. To generate synthetic observations, we incorporated measurement noise and conducted four experiments with varying levels of additive Gaussian noise, set at 2, 5, 10 and 15% of the standard deviation of the data, to check the robustness of our approaches. Figure 2 shows the synthetic observations in red and the model output with $a_{4}=0$ in blue, illustrating clearly that the model consistently underestimates s values. To mimic a real-world scenario in which patients with low SO_2_ values are rarely observed, we have no data points with low values of s .

For this synthetic data study, we used 200 data points for training and 50 data points for validation. When performing the black box method, we represent the model discrepancy term as a zero-mean GP with the squared-exponential correlation function. To avoid the unphysical behaviour of the updated model, f(x)+δ(x) , in the region where s is close to 0 or close to 1, we include our prior knowledge about the model discrepancy in our analysis, similar to [4]. In particular, we condition the process and its derivatives at pre-specified points, i.e. δ(x)=0 and $\delta^{\prime }(x)=0$ with input parameter values that correspond to low and high values of p within the pre-specified range (see appendix (A.3.) for more information). These constraints reflect that δ(x) tends to 0 for small and large values of p , and is exactly zero at these extremes, which is in line with the clinical understanding of how oxygen binds to haemoglobin detailed in §2. Alternatively, we could update a GP model using the information about the model discrepancy behaviour on the boundaries, as proposed by [42] and [43]. We specify an Inverse-Gamma prior for $\sigma^{2}$ with mean $0.3^{2}$ and mode $0.2^{2}$ . Similar to [44], we introduce stronger prior information for p by specifying $\delta_{5}∼\text{Gamma}(4,4)$ , and a smoother prior for the remaining inputs, i.e. $\delta_{i}∼\text{Gamma}(42,9)$ for i=1,…,4 . We also assume that the observational error is fairly well known and choose an Inverse-Gamma prior for $\sigma_{e}^{2}$ with mean $0.016^{2}$ and mode $0.015^{2}$ [4]. We use CmdStanR (Command Stan R) [45] to obtain maximum a posteriori (MAP) estimates for model parameters. We adopt the default optimizer, the limited memory BFGS algorithm [46], to derive these hyperparameters’ values.

These synthetic data are also used to train the grey box model with $a_{4}$ set to be governed by a fully connected neural network with all 5 inputs, 2 hidden layers of 20 neurons each, and a single output representing $a_{4}$ . The activation function used is a simplified form of the radial basis function (RBF), defined as $RBF(x)=e^{-x^{2}}$ . SR was implemented, resulting in a learned mathematical expression representing the trained NN. The model with the learned expression for $a_{4}$ is referred to as the learned model. The unary and binary operators chosen for SR were {sin,cos,ln,e} and {+,−,÷,×} , respectively. The full set of hyper-parameters for the SR implementation are given in table 2 in appendix (A.1.).

**Figure 3.**
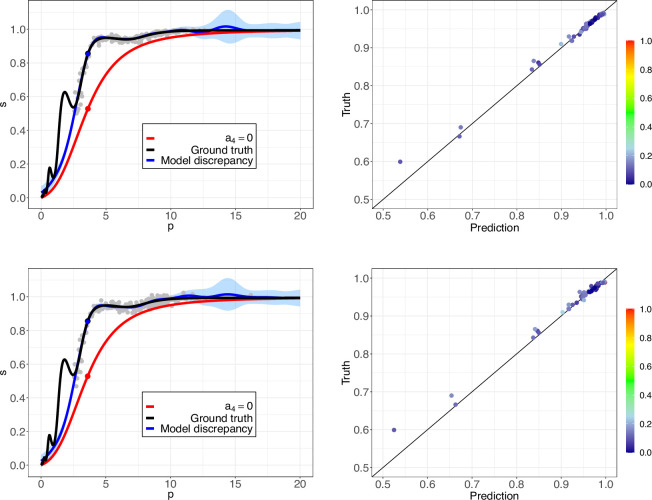
Synthetic data study results. The left plots in Figures (*a*) and (*b*) show the ODC curves for a single patient from the validation set, for 5 and 15% noise. Red curve: S.A. model with $a_{4}=0$ . Black curve: ground truth, where $a_{4}=0.25\;\cos(2\pi \;\ln(p))-0.55$ . The blue curve and grey shaded region correspond to the predictions and two standard deviation prediction intervals obtained with the black box approach. The right plots in figures (*a*) and (*b*) show the ground truth s values, for 5 and 15% noise, against the mean predictions coloured by predictive standard deviations (normalized by their maximum and minimum values).

**Figure 4.**
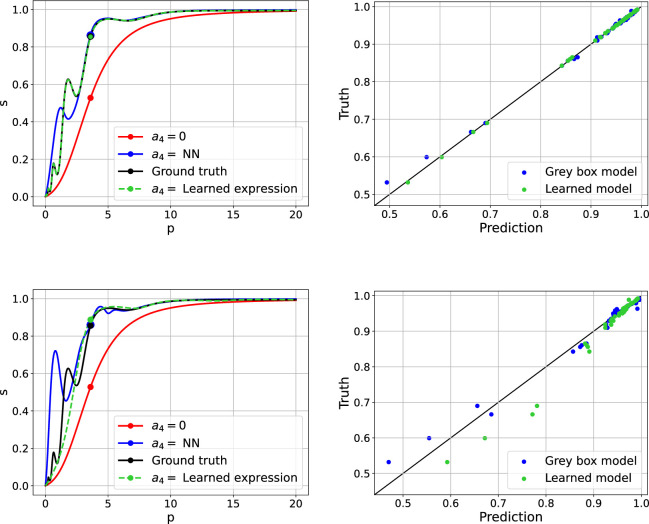
The left plots in figures (*a*) and (*b*) show the ODC curves for a single patient from the validation set, for 5 and 15% noise. Red curve: S.A. model with $a_{4}=0$ . Black curve: ground truth, where $a_{4}=0.25\;\cos(2\pi \;\ln(p))-0.55$ . Blue curve: grey box model, where $a_{4}$ is defined as the trained neural network. Green curve: learned model, where $a_{4}$ is defined as the corresponding expression from table 1. The scatter points represent the predicted s values by each of the models. The right plots in figures (*a*) and (*b*) show the ground truth s values, for 5 and 15% noise, against the predicted s values generated by the grey box model (blue) and the learned model (green), for the validation set.

At each level of noise, we perform diagnostic checks by comparing the ground truth s values against the predictions generated by the two methods. In addition, we select a patient from the validation set in order to compare the corresponding ODCs obtained by these approaches across the different noise levels. The 5 and 15% noise cases are shown for the black box model and the grey box model together with the learned model in figures 3 and 4, respectively. The learned expressions at each level of noise are shown in table 1.

**Table 1.** Learned expressions at each level of measurement noise added to the data. These are the outputs of SR. Note that 2π≈6.2832. all numbers are rounded to four decimal places.

noise	learned expression	true expression
2%	0.2476cos⁡(6.2762ln⁡(p))−0.5477	0.25cos⁡(2πln⁡(p))−0.55
5%	0.2435cos⁡(6.2717ln⁡(p))−0.5495	
10%	0.3074cos⁡(6.3021ln⁡(p))−0.5790	
15%	0.2638cos⁡(p−1.4345)−0.5659	

From the left panel plots in figures 3 and 4, it can be seen that $a_{4}$ is responsible for significant changes in the ODC as the ground truth curve with $a_{4}=0.25\;\cos(2\pi \;\ln(p))-0.55$ (in black) is notably different from the reference curve with $a_{4}=0$ (in red). The black box and grey box models (in blue) perform poorly for lower p values (generally weaker as noise increases), and produce more accurate predictions thereafter. The correct expression structure for $a_{4}$ is recovered for the cases up to and including 10% noise, with deacreasing accuracy in the learned parameter values as noise increases, as shown in table 1. As a result, the predictions of the learned model (in green in figure 4) for these cases are expectedly accurate, with small noticeable deviations from the ground truth for the 10% noise case. For 15% noise, although the correct expression for $a_{4}$ is not found, the corresponding partially learned model outperforms the grey box model, but is still unable to capture the high-frequency variations in the true ODC. This study emphasizes the added benefit of carrying out the inference step using SR, since it can often improve predictions by regularizing the learned model and ‘smoothing’ out oscillations introduced by the high-dimensional NN, particularly in regions where the training data may be sparse (as in the 15% noise case). In this synthetic data study, the reason SR is able improve predictions to this extent is because the form of the correct $a_{4}$ expression is within the function space defined by the unary and binary operators. Contrary to the grey box model and the learned model, the black box approach also produces prediction intervals (grey shaded region), which indicate how confident (certain) we are in the updated model’s predictions. We observe larger prediction intervals for low and high values of p . These intervals can be quite informative and guide us to obtain more data points in these regions to improve the model performance.

For the right panel plots in figures 3 and 4, the model performs well if the predicted values closely align with the true values along the straight 45 ${,}^{\circ }$ line (in black). We plot the predictive mean values against the ground truth s values coloured by normalized predictive standard deviation values at 50 validation data points in figure 3. We observe consistently good performance from the black box approach across all four noise levels considered in the synthetic data study with a few exceptions in the region with low values of s . In figure 4, the grey box model generally predicts more accurately for higher s values, and the overall performance decreases as noise increases. The corresponding learned model shows improvements if the correct expression structure is recovered. The results for the 2 and 10% noise cases are shown in figures 10 and 11 in appendix (A.1.).

## 5. Applications to intensive care unit data

In this section, we proceed to consider the data from an adult ICU together with the haemoglobin-dissociation curve model. Arterial blood gas data are available from 1000 consecutive patients admitted to a single ICU, measured on an ABL90 Flex blood gas analyzer (Radiometer Medical ApS, Denmark). Blood gas values were not corrected for patient body temperature. Despite a large number of recorded values, not all of them contain temperature data, and only 259 records are retained in the present study.

**Figure 5.**
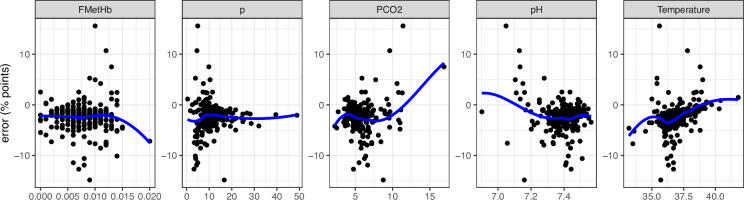
The error in SO_2_ (% points), the difference between observed SO_2_ values and SO_2_ values produced by model with $a_{4}=0$ , against all model inputs with smoothed conditional mean (in blue).

Before performing any model fitting, we apply the transformations given in equations (2.2) and (2.3) to obtain p and s values from the patient’s recorded pO_2_ and SO_2_ values. Figure 5 depicts the difference between the observed SO_2_ and the SO_2_ produced by the mathematical model from §2 with $a_{4}=0$ . The scatter plots highlight the variability in SO_2_ error under different physiological conditions, in particular we tend to observe larger deviations in model predictions from observations for lower values of p and pH.

Similar to the synthetic data study in §4, we consider the mathematical model of the haemoglobin-dissociation curve with a single output s and the following inputs (pH, PCO_2_, T , p , MetHb). To communicate the results more clearly in a clinical setting, we subsequently convert the predicted s values to SO_2_ values. In this study, we specify a zero-mean GP with a squared exponential covariance function to represent the model discrepancy term. The observational error is negligible in this study, since following the clinical expertise the patient’s records of SO_2_ are highly accurate. We use the MAP method with reference priors in the R package RobustGaSP to obtain the model parameters [47]. For the grey box model, the $a_{4}$ term is defined as an NN with 5 inputs, 2 hidden layers of 64 neurons each and the exponential linear unit (ELU) activation function, defined in appendix (A.2.). To obtain the learned model, we use SR with the same hyper-parameters as in §2. We perform 10-fold cross-validation to assess the performance of our methods.

**Figure 6.**
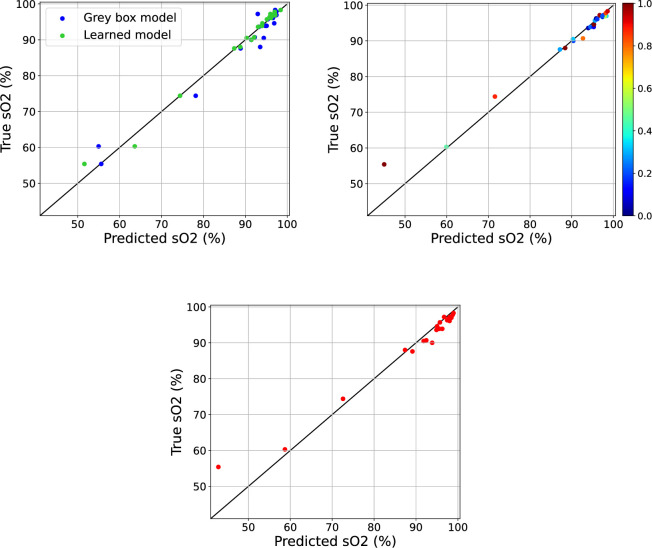
Top left panel: predicted SO_2_ values against the true SO_2_ measurements for grey box model (blue) and learned model (green). Top right panel: predictive mean values of SO_2_ against the true SO_2_ measurements coloured by predictive standard deviations (normalized by their maximum and minimum values). *Bottom panel:* predicted SO_2_ values from the original S.A. model with $a_{4}=0$ against the true SO_2_ measurements.

**Figure 7.**
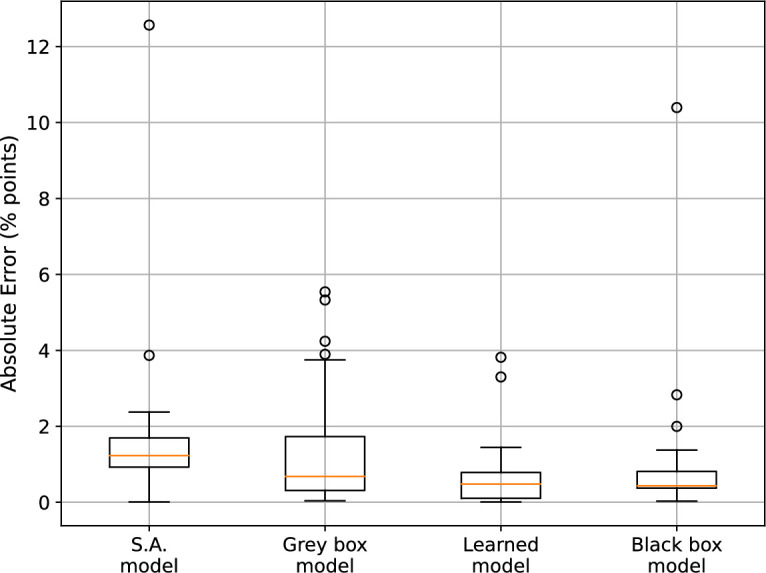
Comparison of absolute error (in percentage points) across different model types.

Figure 6 shows the predicted ${\text{SO}}_{2}$ values against the measured (‘true’) ${\text{SO}}_{2}$ values for the fold 10 validation set. The corresponding results for the remaining folds are shown in figures 12–20. Figure 6*a* depicts the results for the grey box model and the learned model from the SciML approach. The learned expression for $a_{4}$ for this fold is 0.03346p×pH−0.00592p×T−0.1786 , which is interesting since it is in line with the clinical understanding that the impact of 2,3-DPG varies with changes in pH and temperature [28]. The learned expressions for all folds are given in table 3 in appendix (A.2.), and we can see that pH and temperature are consistently selected in the learned expression by SR. Figure 6*b* illustrates the results for the black box approach. From figure 6*a*,*b* we can observe that all three approaches perform better for higher values of SO_2_ with predictions being closer to the observed value where more data are available. In general, this is also true for the other folds with a few exceptions. For comparison, figure 6*c* shows the results for the S.A. model with $a_{4}=0$ . Figure 7 shows the distribution of the absolute errors of each of the models for the fold 10 validation set. All three approaches outperform the S.A. algorithm (where $a_{4}=0$ ) in terms of the lower median absolute error (in orange), with the grey box model being the only one with a greater interquartile range than the S.A. model. For this particular fold, the learned model performs the best when considering the outliers, however this is not always the case. Box plots showing the absolute error distribution of the models for the remaining folds are also shown in figures 12–20 in appendix (A.2.)

**Figure 8.**
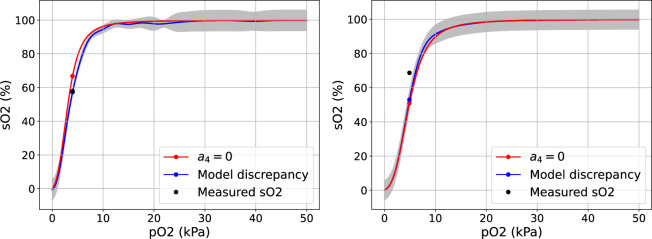
ODC curve generated by the S.A. model (red), the blue curve and grey shaded region correspond to the predictions and two standard deviation prediction intervals obtained with the black box approach for two patients from the test set of different folds. The black scatter point shows the measured ${\text{SO}}_{2}$ value and the coloured scatter points show the corresponding predicted ${\text{SO}}_{2}$ values by each model.

**Figure 9.**
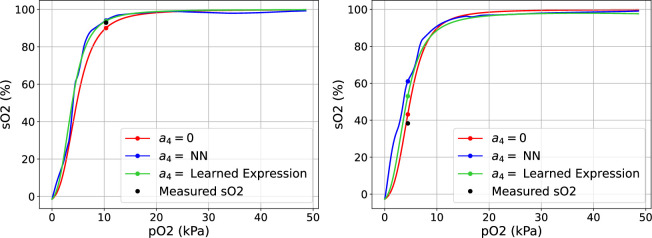
ODC curves generated by the S.A. model (red), grey box model (blue) and corresponding learned model (green) for two patients from the test set of different folds. The black scatter point shows the measured ${\text{SO}}_{2}$ value and the coloured scatter points show the corresponding predicted ${\text{SO}}_{2}$ values by each model.

We also choose to demonstrate the ODCs obtained through our approaches. For each method, we selected two observed records (patients) from the validation datasets across all folds, based on the absolute improvements over the S.A. model with $a_{4}=0$ : one with a high score and one with a low score. Figures 8 and 9 show the predicted ODC curves for two patients. From figure 8*a*, we can observe that the predicted curve outperforms the S.A. model with predicted SO_2_ value (mean: 57.2% and s.d.: 0.9%) close to the observed value (57%). The SO_2_ value reported by the S.A. model is 66.8%. Figure 8*b* shows that the ODC curves obtained by the S.A. model and black box approach overlap and produce SO_2_ values (mean: 53% and s.d.: 2.65% versus 51%) well below the observed SO_2_ value (68.7%). Figure 9*a* shows the predicted curves for a patient with a high improvement score, demonstrating how the predicted SO_2_ values by grey box model (94.4%) and the learned model (93.2%) outperform the S.A. model (90.0%) to generate curves that are closer to the observed ${\text{SO}}_{2}$ value (92.9%). Figure 9*b* shows the predicted curves with a low improvement score, which do not perform as well, highlighting the issue of the lack of data for low pO_2_ values. The SO_2_ value generated by the S.A. model (43.2%) is closer to the observed value (38.3%) than the SO_2_ values predicted by the grey box model (61.3%) and the learned model (53.0%), which are significantly higher.

## 6. Discussion

The aim of intensive care is to support patients during definitive treatment or recovery, without causing additional harm. In the last 30 years, intensive care physicians have moved from a paradigm of ’normalizing to abnormal’ to supporting adaptive physiology, which largely aligns with the principles of precision medicine. Part of this paradigm shift has evolved from the realization that many intensive care interventions can cause harm with the adverse effects of overtreatment with oxygen being increasingly recognized [48]. Treatments targeting oxygen delivery could be optimized by recognizing and responding to haemoglobin oxygen affinity in addition to the measured SO_2_. The haemoglobin oxygen affinity is difficult to measure in practice, and in this paper we considered the S.A algorithm, commonly used in ICU settings to provide affinity-based estimates.

This mathematical model suffers from model inadequacy, and in this paper we have presented methods from UQ and SciML to address this issue. We assessed the performance of these approaches with a synthetic data study, where SR showed an impressive capability of recovering the true $a_{4}$ expression for the cases up to and including 10% noise. The prediction intervals generated by the black box approach in this study can provide insights into the future data collection process. For the clinical data study, while the grey box and learned models outperform the S.A. algorithm the majority of the time, both approaches tend to occasionally underperform in the regions with low pO_2_ and SO_2_ values, since most of the provided clinical data are in the arterial range. To address this issue in future work, data from venous blood gases, which are measured clinically, but less frequently than arterial blood gases, can be used in our analysis and may improve our estimation of the lower part of the ODC. In addition, blood gases from those chronically adapted to hypoxia, for example, those who live at high altitude, or those with cyanotic heart disease may provide valuable information for this lower part of the ODC. While haemoglobin oxygen affinity is difficult to measure, experimental set-ups can be used to directly measure this to validate our estimations of the ODC. Recently, collaborators have described a set-up to measure single red blood cell oxygen saturations, and the capacity for oxygen release [48]. We are aiming to generate data from experiments with patient blood samples to emulate low oxygen conditions and improve estimations of the whole ODC.

Traditionally, UQ methods have been developed for computationally expensive mathematical models in physical sciences and engineering [49]. Therefore, treating these models as black box systems is common, with model discrepancy typically modelled as an additive, independent term accounting for limitations in model representation of the physical process of interest. On the contrary, the scientific machine learning approach allows the discrepancy arising from specific model components to be targeted, while retaining the remaining equation structure, which can significantly help modellers at the model development stage. Recovering mathematical expressions for the targeted components via the SR step provides insight into the system, which can be crucial in a clinical setting, where a clear understanding of a model output that may influence treatment decisions is vital. While the ability of SR to learn interpretable expressions can be very beneficial, its utility can be limited when modelling real-world phenomena, where data are noisy and missing model components may not have simple closed forms. Despite this, the use of SR in these settings is still good practice, given that mathematical equations that govern physical laws are often parsimonious and very accurate in describing real-world phenomena. However, this approach does not account for the uncertainty in the model predictions, which is another important metric to consider in clinical decision-making. Additionally, since the scientific machine learning method targets specific components of a model, the remaining structure of the model is often assumed to not contribute to the overall model uncertainty, as was also assumed in this work—a potential limitation that should be considered when interpreting the results. Researchers in the UQ and SciML fields could greatly benefit from close collaborations. In particular, when operating with observational data and partially known mathematical models, SciML methods could benefit from careful treatment of major sources of uncertainties commonly studied in the UQ field, while adding explainable ML approaches such as SR to the UQ arsenal could help with interpretability of results.

## Figures and Tables

**Figure 10.**
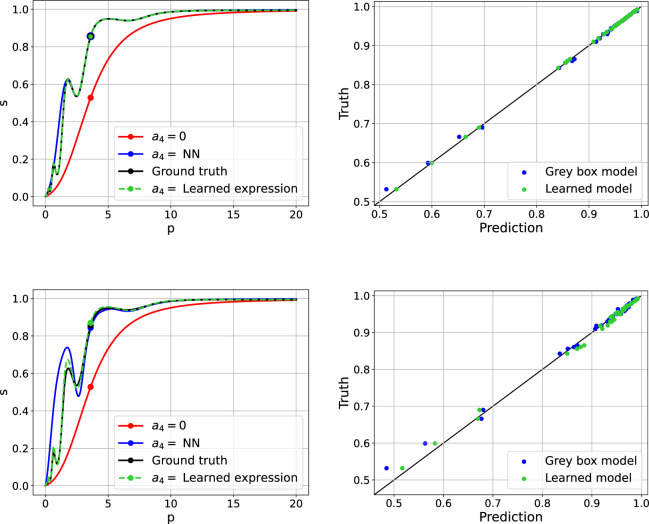
Synthetic data study results. The left plots in Figures (*a*) and (*b*) show the ODC curves for a single patient from the validation set, for 2 and 10% noise. Red curve: S.A. model with $a_{4}=0$ . Black curve: ground truth, where $a_{4}=0.25\;\cos(2\pi \;\ln(p))-0.55$ . Blue curve: grey box model, where $a_{4}$ is defined as the trained neural network. Green curve: partially learned model, where $a_{4}$ is defined as the corresponding expression from table 1. The right plots in Figures (*a*) and (*b*) show the ground truth s values, for 2 and 10% noise, against the predicted s values generated by the grey box model (blue) and the learned model (green), for the validation set.

**Figure 11.**
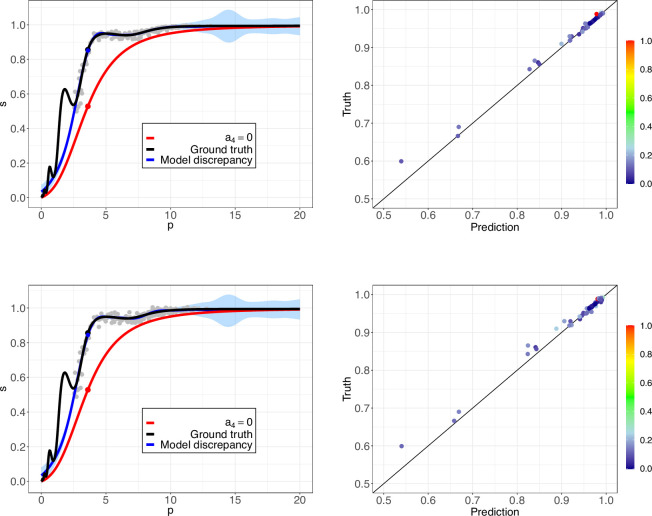
Synthetic data study results. The left plots in figures (*a*) and (*b*) show the ODC curves for a single patient from the validation set, for 5 and 15% noise. Red curve: S.A. model with $a_{4}=0$ . Black curve: ground truth, where $a_{4}=0.25\;\cos(2\pi \;\ln(p))-0.55$ . The blue curve and grey shaded region correspond to the predictions and two standard deviation prediction intervals obtained with the black box approach. The right plots in figures (*a*) and (*b*) show the ground truth s values, for 5 and 15% noise, against the mean predictions coloured by predictive standard deviations (normalized by their maximum and minimum values).

**Figure 12.**
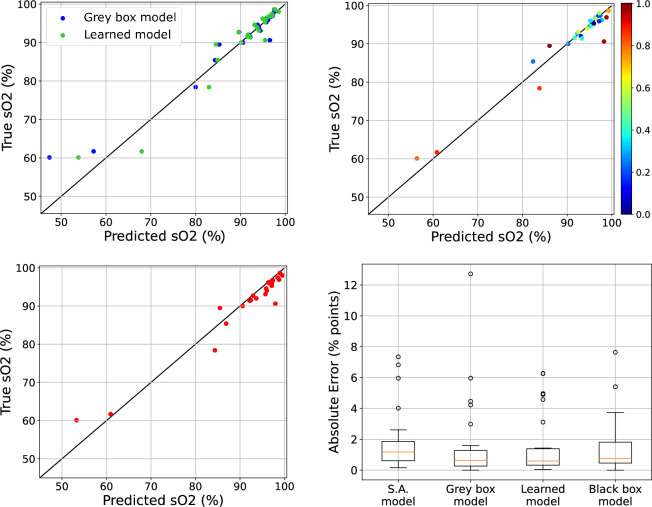
Comparison of model performance on fold 1. *Top row:* predicted SO_2_ values against the true SO_2_ measurements for grey box model (blue) and learned model (green) (*left panel plot*). The predictive mean and two standard deviation prediction intervals produced by the black box approach are in black. The true SO_2_ measurements are in green if they lie within two standard deviation prediction intervals, or red otherwise (*right panel plot*). *Bottom row:* comparison of absolute error (in percentage points) across different model types.

**Figure 13.**
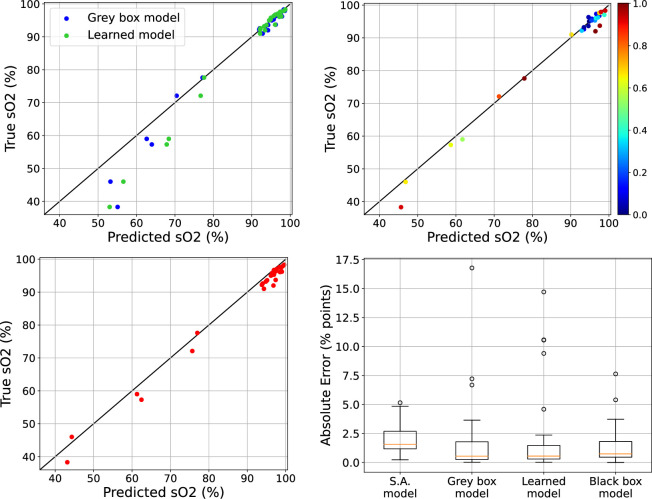
Comparison of model performance on fold 2.

**Figure 14.**
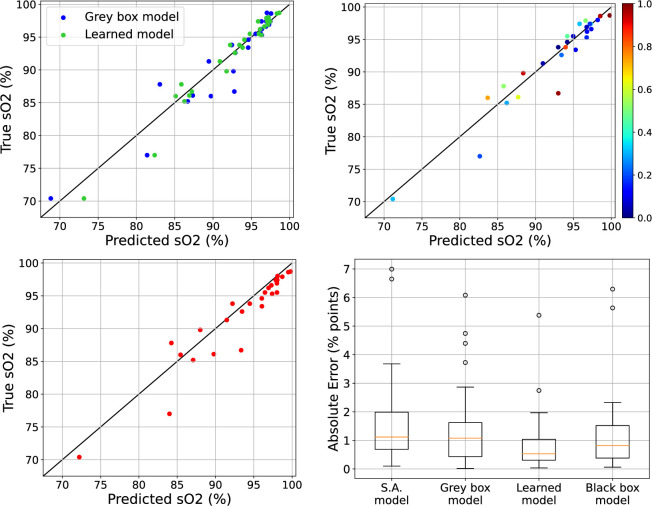
Comparison of model performance on fold 3.

**Figure 15.**
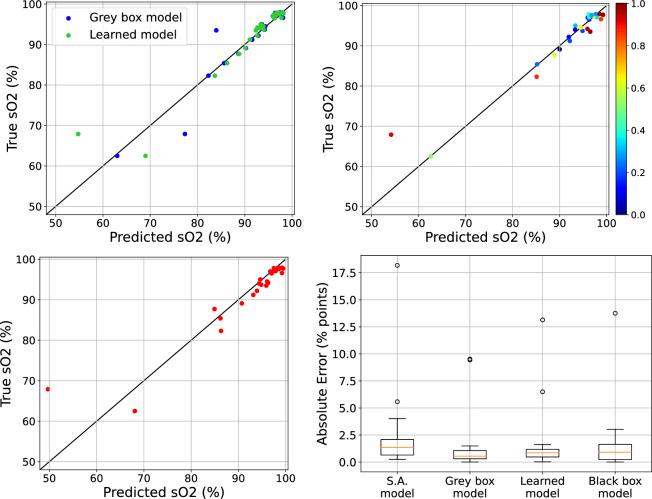
Comparison of model performance on fold 4.

**Figure 16.**
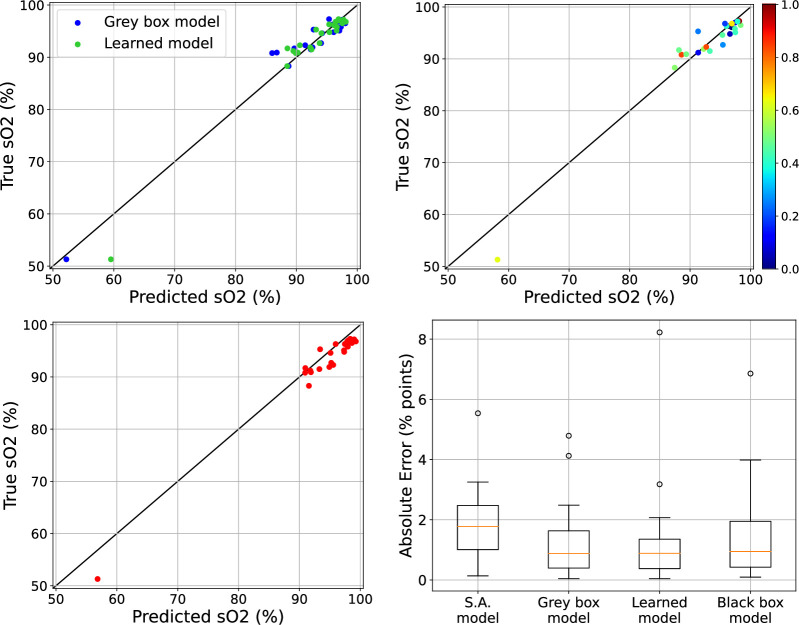
Comparison of model performance on fold 5.

**Figure 17.**
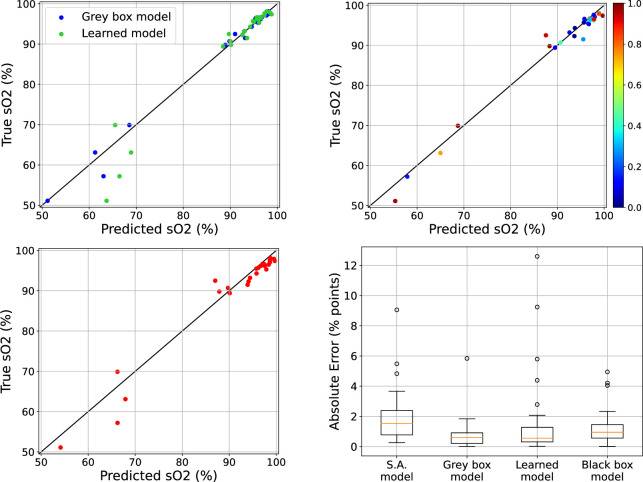
Comparison of model performance on fold 6.

**Figure 18.**
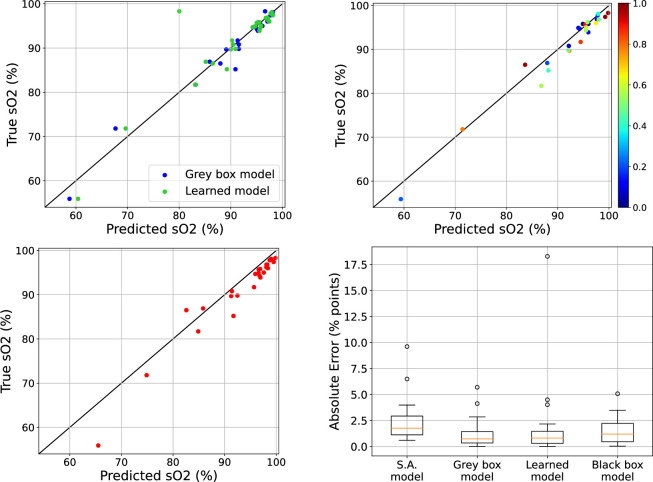
Comparison of model performance on fold 7.

**Figure 19.**
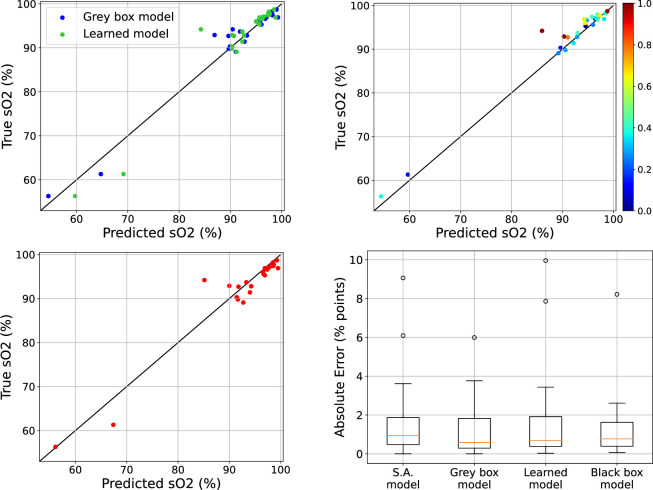
Comparison of model performance on fold 8.

**Figure 20.**
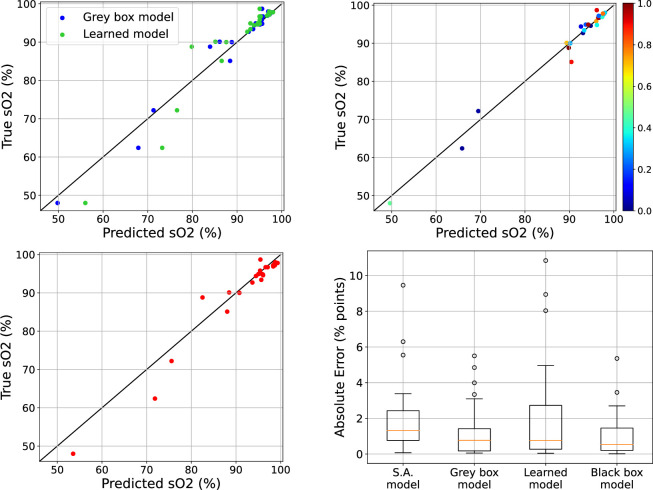
Comparison of model performance on fold 9.

## Data Availability

The code and data used in this work are available from the Zenodo digital repository [50].

## A. Appendix

### A.2. Applications to intensive care unit data study

ELU activation function used in clinical data study with α=1.0 : figures 10 and 11, table 2.

(A 1)
$$\begin{array}{r} \text{ELU}(x)=\left\{ \begin{array}{ll} x & \text{if }x\geq 0,\\ \alpha (\exp(x)-1) & \text{if }x<0, \end{array}\right. \end{array}$$

**Table 2.** PySR hyper-parameters.

unary operators	{sin,cos,ln,e}
binary operators	{+,−,÷,×}
functions per population	33
populations	400
iterations	200
performance metric	MSE

### A.3. Constrained GP prior

We demonstrate how to incorporate prior knowledge about the model discrepancy in our analysis. We note that the derivatives of the GP are also a GP [51]. For the first-order derivative of δ(x) , a zero mean GP with squared exponential covariance function, defined in §3, we can write down: table 3.

(A 2)
$$\begin{array}{rl} & E[\frac{\partial \delta (x)}{\partial x^{\left(k\right)}}]=0 \end{array}$$

(A 3)
$$\begin{array}{rl} & C[\frac{\partial \delta (x)}{\partial x^{\left(k\right)}},\delta (x^{\prime })]=k^{10}(x,x^{\prime })=-\sigma^{2}\frac{2(x_{k}-x_{k}^{\prime })}{\delta_{k}^{2}}\exp\;\{-\sum_{l=1}^{p}(\frac{x_{l}-x_{l}^{\prime }}{\delta_{l}}{)}^{2}\} \end{array}$$

(A 4)
$$\begin{array}{rl} & C[\frac{\partial \delta (x)}{\partial x^{\left(k\right)}},\frac{\partial \delta (x^{\prime })}{\partial x^{\prime \left(k\right)}}]=k^{11}(x,x^{\prime })=\frac{2\sigma^{2}}{\delta_{k}^{2}}\exp\;\{-\sum_{l=1}^{p}(\frac{x_{l}-x_{l}^{\prime }}{\delta_{l}}{)}^{2}\}\;(1-2(\frac{x_{k}-x_{k}^{\prime }}{\delta_{k}}{)}^{2}), \end{array}$$

where k indicates which input the derivative is with respect to.

**Table 3.** Learned expressions for $a_{4}$ via SR for each fold of the cross-validation. All numbers are rounded to four decimal places.

fold	learned $a_{4}$ expression
1	0.3045pH−0.0660T+0.0292p
2	0.3357pH−0.0724T+0.0289p
3	0.0405pH−0.0075p×T−0.1291
4	0.3397pH−0.0730T+0.0290p
5	0.3129pH−0.0677T+0.0288p
6	0.2677pH−0.0589T+0.0294p
7	$0.3216\text{pH}-0.0641T+0.0009p^{2}$
8	0.0999pH−0.0238T+0.02382p
9	0.2979pH−0.0648T+0.0295p
10	0.03346p×pH−0.0059p×T−0.1786

Consider the prior information about the model discrepancy $\delta_{c}=\left(\delta (S),\delta^{\prime }(S)\right)$ , where S is the collection of input parameters of size m=32 , designed using a factorial design for four variables (pH, PCO_2_, FMetHb and T ), where each variable set to its respective minimum and maximum values and with p set at values close to 0 and 20. We can write down the joint distribution for observation vector z and $\delta_{c}$ as

$$\begin{array}{rlr} \left(\begin{array}{c} z\\ \delta_{c} \end{array}\right)|\sigma_{e}^{2},\sigma^{2},\gamma & ∼ & \text{N}\left[\left(\begin{array}{c} F\\ 0 \end{array}\right),\left(\begin{array}{cc} \sigma_{e}^{2}I+K & (K^{10}{)}^{T}\\ K^{10} & K^{11} \end{array}\right)\right], \end{array}$$

where $F=\left(f(x_{1}),\ldots ,f(x_{n})\right)$ , I is an n×n identity matrix, and K is an n×n matrix with entries $K_{ij}=k(x_{i},x_{j})$ . In addition, we specify $K^{10}$ as a 2m×n matrix with entries:

$$K_{ij}^{10}=\left\{ \begin{array}{cc} & k(s_{i},x_{j}),\;i=1,2,\ldots ,m,j=1,\ldots ,n\\ & k^{10}(s_{i-m},x_{j}),\;i=m+1,\ldots ,2m,j=1,\ldots ,n, \end{array}\right.$$

and $K^{11}$ as a 2m×2m covariance matrix with entries:

$$K_{ij}^{11}=\left\{ \begin{array}{cc} & k(s_{i},s_{j}),\;i=1,2,\ldots ,m,j=1,2,\ldots ,m\\ & k^{11}(s_{i-m},s_{j-m}),\;i=m+1,\ldots ,2m,j=m+1,\ldots ,2m\\ & k^{10}(s_{i-m},s_{j}),\;i=m+1,\ldots ,2m,j=1,\ldots ,m. \end{array}\right.$$

By performing the conditioning in multivariate normal distribution, we can obtain the distribution for z given $\delta_{c}$ and model parameters:

(A 5)
$$\begin{array}{lcr} & z|\delta_{c},\sigma_{e}^{2},\sigma^{2},\gamma ∼\text{N}[F,\sigma_{e}^{2}I+K-(K^{10}{)}^{T}(K^{11}{)}^{-1}K^{10}], & \end{array}$$

which we use as our model likelihood when performing Bayesian inference.
